# Second annual report from the ISSPP PIPAC database

**DOI:** 10.1515/pp-2023-0047

**Published:** 2023-12-13

**Authors:** Michael Bau Mortensen, Francesco Casella, Özgül Düzgün, Olivier Glehen, Peter Hewett, Martin Hübner, Magnus Skov Jørgensen, Alfred Königsrainer, Miguel Marin, Marc Pocard, Günther Rezniczek, Jimmy So, Claus Wilki Fristrup

**Affiliations:** Upper GI and HPB Section, Department of Surgery, Odense Universitetshospital, Odense, Denmark; General and Upper GI Surgery, University of Verona, Verona, Veneto, Italy; Department of Surgical Oncology, İstanbul Ümraniye Training and Research Hospital, Istanbul, Türkiye; Service de Chirurgie Digestive et Oncologique, Hôpital Lyon Sud, Hospices Civils de Lyon, Lyon, France; Department of Surgery, The Queen Elizabeth Hospital, Woodville South, South Australia, Australia; Lausanne University Hospital CHUV, University of Lausanne, Lausanne, Switzerland; Department of Surgery, Odense University Hospital, Odense, Denmark; Department of General, Visceral and Transplant Surgery, University Hospital Tübingen, Tübingen, Germany; Department of Surgery, Reina Sofia University General Hospital, Murcia, Spain; Surgical Unit, Paris 7 University, Paris, France; Obstetrics & Gynecology, Ruhr-Universität Bochum, Herne, Germany; Surgery, National University Hospital, Singapore, Singapore

**Keywords:** database, ISSPP, PIPAC, peritoneal metastasis

## Abstract

**Objectives:**

To monitor the results of PIPAC directed therapy based on data from the International Society for the Study of the Pleura and Peritoneum (ISSPP) PIPAC database.

**Methods:**

Analysis of data from patients entered between June 15th, 2020, and February 28th, 2023.

**Results:**

Twelve centers reported 2,456 PIPAC procedures in 809 patients (median 2, range 1–18) with peritoneal metastasis (PM) from different primary tumors. Approximately 90 % had systemic chemotherapy prior to PIPAC. Twenty-eight percent were treated in prospective protocols. Overall non-access rate was 3.5 %. Concomitant surgical procedures were performed during PIPAC in 1.6 % of the patients. Median length of stay was 2 days. A total of 95 surgical complications were recorded, but only 22 % of these were graded ≥3b. Seventeen-hundred-and-three adverse events were noted, and 8 % were classified ≥3. The rate of complete or major histological response (peritoneal regression grade score, PRGS≤2) increased between the first and the third PIPAC in the group of patients who were evaluated by PRGS, and a PRGS ≤2 or a reduction of the mean PRGS of at least 1 between first and third PIPAC were observed in 80 %. Disease progression (50 %) or technical issues (19 %) were the most important reasons for stopping PIPAC treatment. Median overall survival from first PIPAC directed treatment varied from 10.7 months (CI 8.7–12.5) in gastric cancer to 27.1 months (16.4–50.5) in mesothelioma.

**Conclusions:**

The ISSPP PIPAC database provides substantial real-world data supporting the use of PIPAC directed therapy in patients with PM from different primary tumors.

## Introduction

Pressurized intraperitoneal aerosol chemotherapy (PIPAC) directed therapy has been used for the treatment of unresectable peritoneal metastasis since its first use in humans in 2011 [1]. Substantial research efforts on dose-finding, safety profile, response evaluation, and subsequent prospective phase II trials have provided insight into the oncological potential of this new treatment platform. However, PIPAC directed therapy has not yet reached its final maturity due to lack of randomized trials, and PIPAC is still considered experimental. Having reached stage 2b of the IDEAL recommendations [2] a prospective international PIPAC registry group and database were advocated by the International Society for the Study of the Pleura and Peritoneum (ISSPP) Executive Committee. An international PIPAC database was officially launched by ISSPP in June 2020. The design, access, implementation process, and preliminary data analysis from the database was published in 2021 [3].

In accordance with the ISSPP Registry Group articles of association, we now provide the second annual report. Apart from providing an updated overview of the reported data, this report may also contribute to the continuing process of updating and changing of variables, database usability and inclusion of new PIPAC centers.

## Materials and methods

This report concerns data entered into the ISSPP PIPAC online database between the official launch on June 15th, 2020, and February 28th, 2023. Software, hosting, governance, legal aspects, ethical framework, variables, and implementation of the database have been described previously [3].

### Statistics

Descriptive data was presented as numbers and percentage for categorical variables and as median or mean for continuous variables. Differences between groups were tested by χ^2^-test for categorical variables and Mann–Whitney-U-tests and Fisher’s exact test for continuous variables. Survival data was evaluated using the Kaplan–Meier approach and log rank test. Statistical significance was considered at a two-sided p-value ≤0.05.

### Financial aspects

The ISSPP PIPAC database is funded and hosted by Open Patient data Explorative Network (OPEN), Odense University Hospital, Odense, Denmark, in close collaboration with Odense PIPAC Center (OPC). Maintenance, inclusion of new centers, change of variables, reminders to including centers, and data analysis are performed by the ISSPP PIPAC Research Project Manager (CWF) supported by the ISSPP Registry Group chairman (MBM) and the ISSPP Registry Group. The work of the Research Project Manager was supported by an annual grant from the ISSPP until January 2022. Since then, the ISSPP PIPAC database has been solely funded by OPC and OPEN.

## Results

Twelve centers reported data on 809 patients treated by PIPAC directed therapy in the ISSPP PIPAC Database between June 2020 and March 2023. The number of included patients ranged from 2–355 (median 10), but four centers provided 93 % of the included patients. Demographic data are listed in Table 1. Gastric, colon and ovarian cancer accounted for 2/3 of the included patients. Seventy percent of the patients were diagnosed with peritoneal metastasis (PM) within 6 months of their primary tumor, and 89 % received oncological treatment prior to their first PIPAC directed therapy.

**Table 1:** Demographic data.

No. patients	809
Age, years (range)	61 (22–87)
Female/male	435/374
ECOG performance status	0: 26.7 %
	1: 59.7 %
	2: 5.8 %
	3: 0.1 %
	N.A. 7.7 %

Primary tumor, n (%)	

Gastric	287 (35)
Colon	155 (19)
Ovaries	96 (12)
Appendix	62 (8)
Pancreas	54 (7)
Mesothelioma	45 (6)
Bile duct	28 (3)
Small bowel	18 (2)
Rectum	10 (1)
Esophagus	6 (0.7)
Breast	5 (0.6)
Primary peritoneal	4 (0.5)
Uterus	2 (0.2)
Fallopian tube	1 (0.1)
Unknown	3 (0.4)
Other	33 (4)

Primary tumor, *in situ*	61 %

Histology, primary tumor	Adeno 45 %
	Mucinous adeno 5 %
	Signet-ring-cell carcinoma 24 %
	Ovarian 7 %
	Pseudomyxoma peritonei 2 %
	Mesothelioma 5 %
	Other/N.A. 12 %
Time from diagnosis of primary to PM	0–2 months: 66.3 %
	3–5 months: 4.3 %
	≥6 months: 24.4 %
	Unknown/N.A.: 5.0 %

Extra-peritoneal metastases	6.8 %

Previous oncological treatment	Yes 88.7 %
Type of treatment (patients may receive several different treatments)	Systemic chemo 88.0 %
	Immunotherapy 2.2 %
	Radiotherapy 2.6 %
	Other 4.8 %

### PIPAC procedures

A total of 2,456 PIPAC procedures were performed (Table 2). The overall non-access rate was 3.5 %, and this rate dropped significantly from 7.8 % at first PIPAC to 1.5 % during subsequent PIPACs (p<0.01). Two-thirds of the patients had bi-directional treatment, when this was defined as systemic chemotherapy within 4 weeks prior to PIPAC directed treatment. This interval did not change significantly between PIPAC 1–3 and PIPAC 4+ (p=0.089).

**Table 2:** PIPAC procedures.

Total number of procedures	2,456
Number of PIPAC per patient, median (range)	2 (range 1–18)
Ascites, reported cases and median volume (range)	N=911, 200 mL (0–19,000 mL)
PCI complete registration at first PIPAC (all procedures)	76 % (69 %)
PCI score at first PIPAC, median (range)	18 (0–39)
PCI score, all procedures, median (range)	19 (0–39)
Electrostatic precipitation	6.5 %
Other surgical procedures during PIPAC	38 (1.6 %)
Median length of stay (95 % percentile)	2 days (4 days)

PCI, Peritoneal Cancer Index.

Additional surgical procedures were performed during PIPAC in 1.6 % of the patients, only. These procedures included bowel resection and/or suturing (18 %), adnexectomy (15 %), parietal peritonectomy (13 %), omentectomy (5 %) and other/N.A. (49 %).

The reported doses of chemotherapy, flow rates and exposure time are listed in Tables 3 and 4. The median length of stay was 2 days for the first six PIPAC procedures and 1 day for additional procedures. Only 27.7 % of patients facing their first PIPAC directed treatment did so as part of a prospective study.

**Table 3:** Reported doses of oxaliplatin, cisplatin and doxorubicin during first PIPAC treatment (n=766).

Drug (n)	Doses	n
Oxaliplatin (194)	<90 mg/m^2^	2
	90 mg/m^2^	68
	92 mg/m^2^	90
	≥92.5 mg/m^2^	34
Cisplatin (564)	<7.5 mg/m^2^	2
	7.5 mg/m^2^	175
	10.5 mg/m^2^	365
	>10.5 mg/m^2^	22
Doxorubicin (564)	1.5 mg/m^2^	175
	2.1 mg/m^2^	363
	Other	26
Mitomycin (8)		8
Nab-paclitaxel (0)		0
Other (0)		0

**Table 4:** Flow rates and exposure time.

Flowrate, mL/s (n=408)	n (%)
0.4–0.59	187 (45.8)
0.6–0.79	145 (35.5)
0.8–0.99	64 (15.7)
1.0-	12 (2.9)

**Exposure time, minutes (n=768)**	

1	28 (3.7)
6	22 (2.9)
12	25 (3.3)
30	693 (90.2)

### Complications and adverse events

A total of 95 surgical complications were recorded during 2,456 PIPAC procedures. Twenty-one (22 %) of these were ≥3b according to Dindo–Clavien (Table 5). No patient or procedure was linked to more than one grade 3b complication. There was no statistical difference in the rate of surgical complications among the different primary tumors.

**Table 5:** Complications and adverse events.

Total number of procedures	2,456
Number of detailed and graded event	Surgical: 95
	Adverse events: 1,703
Surgical complications	Grade 1–3a: 74 (78 %) (bleeding, access lesion, bowel lesion, ascites fistula, other^a^)
	Grade 3b: 19 (20 %) (bowel injury, perforation, other^a^)
	Grade 4a: 2 (2 %) (other^a^)
Adverse events	Grade 1: 637 (37 %)
	Grade 2: 932 (55 %)
	Grade 3: 101 (6 %)
	Grade 4: 28 (1.5 %)
	Grade 5: 5 (0.5 %)
Most common adverse events, n	Abdominal pain 468
	Nausea 286
	Vomiting 211
	Constipation 156
	Urinary retention 106
	Peripheral sensory neuropathy 69
	Ileus 54
	Diarrhea 43

^a^n=14, 12 port site hemorrhage, hematoma or herniation, 2 misclassifications.

Seventeen-hundred-and-three adverse events (AE) were registered according to CTCAE (Table 5). Twenty-five procedures were linked with two AE grade ≥3, and 6 procedures with 3 AE grade ≥3. Abdominal pain, nausea, vomiting, constipation and urinary retention were most frequent AE (72 %). Overall 134 (8 %) AE were graded ≥3. Postoperative mortality was zero percent (0/669) on the day of the last PIPAC procedure, while 30 days mortality was 3.4 % (23/669) (CI 2–5 %). The exact date of death was missing in 76 patients who were recorded as deceased, and these patients were not included in the calculation.

### Response evaluation

Response to PIPAC directed treatment was reported after 2,115 procedures (peritoneal regression grade score, PRGS, n=1,980 (93.6 %), non-PRGS n=135 (6.4 %)). Response evaluation was not available in 83 % of the non-PRGS group. Complete, partial and no response were seen in 5 , 10 and 85 % of the remaining patients (n=20). Overall mean PRGS dropped from 2.2 at first PIPAC to 1.8 after the third PIPAC, and 1.6 after the sixth PIPAC. The rate of complete or major histological response (PRGS≤2) increased between the first and the third PIPAC in the group of patients who were evaluated by PRGS (Table 6). A PRGS ≤2 or a reduction of the mean PRGS of at least 1 between first and third PIPAC were observed in 80 % (n=309).

**Table 6:** Rate of patients with complete/major response (PRGS≤2) at first, second and third PIPAC according to primary tumor (stomach, colon, ovaries, appendix or pancreas).

	PIPAC 1 (n)	PIPAC 2 (n)	PIPAC 3 (n)
Stomach	75.1 % (130)	81.1 % (116)	83.3 % (100)
Colon	66.4 % (71)	87.8 % (65)	93.2 % (55)
Ovaries	49.2 % (31)	59.1 % (26)	71.4 % (25)
Appendix	50.0 % (13)	85.2 % (23)	85.7 % (18)
Pancreas	59.5 % (22)	74.1 % (20)	82.4 % (14)

### Ascites

The presence of ascites was noted during 911 procedures with a median volume of 200 mL (range 0–19,000 mL). The number of patients with >500 mL of ascites dropped significantly between PIPAC 1–3 and PIPAC 4+ (p<0.01), and the same was observed for patients presenting with >1,000 mL of ascites (p<0.01).

### Survival

The median overall survival from first PIPAC directed therapy was 12.8 months (n=752, 95 % CI 11.5–13.8). Survival according to primary tumor is shown in Table 7.

**Table 7:** Median overall survival from first PIPAC directed treatment according to primary tumor, where at least 15 patients were treated (n=728).

Primary tumor	n	Median overall survival, months (95 % CI)
Stomach	269	10.7 (8.7–12.5)
Colon	140	16.0 (11.7–22.1)
Ovaries	95	12.1 (8.6–17.3)
Appendix	58	18.4 (13.2–37.2)
Pancreas	50	11.5 (7.9–13.9)
Mesothelioma	43	27.1 (16.4–50.5)
Bile duct	23	10.0 (5.6-)
Small bowel	17	10.9 (7.3–13.5)
Other	33	12.5 (9.8–29.6)

The median observation time for patients alive was 8.2 months (range 0–74 months), but three out of the four largest centers had a median observation time of at least 11 months. Follow up validity when measured by true registration of death was 71 % (range 0–100).

### PRGS and survival

A comparison of survival from first PIPAC procedure between patients with and without evidence of local tumor response was only possible for patients with gastric cancer due to lack of recorded follow up events for patients with other primary tumors. Patients with gastric cancer (n=117) who had a PRGS of ≤2 at PIPAC 1 and 3 and/or a drop in PRGS of at least 1 between PIPAC 1 and 3 had a statistical significant better median survival (13.6 vs. 8.2 months, p=0.006) than those without this response. Patients with colon cancer (n=52) and response also had a better survival – however not statistically significant (21.2 vs. 14.7 months, p=0.059).

### Reasons for stopping PIPAC

Information on reasons for stopping PIPAC was available for 84 % of the patients. The major cause was disease progression (50 %), technical reasons (19 %), patient refusal (6 %) and other (not specified) in 5 %. End of study was the reason in 2.5 % and curative intended surgery in 1.4 %. Less than 0.5 % was due to lost to follow up, and no reason(s) for stopping PIPAC was provided in 16 %.

## Discussion

The present data were registered in the ISSPP PIPAC Database over a period of 32 months (on 809 patients), and included data form the first 6 months (on 181 patients) which has previously been reported [3].

Although not directly comparable, analysis of the present data from the ISSPP PIPAC Database seems to match results from recent narrative and extensive systematic reviews and meta-analysis of patients undergoing PIPAC directed therapy [4, 5]. Variations regarding intraoperative settings like flow rate, exposure time, drugs and doses merely reflect experience and changes over time based on ongoing and completed trials, new aerosolizers, and updated PIPAC recommendations [5], [6], [7], [8].

The results of several of the outcome variables suggest that most patients were well selected for PIPAC directed therapy, and that repeated PIPAC directed treatments were possible with the expected low rate of significant complications and adverse events. Other variables including non-access rate, length of stay, number of PIPAC procedures, bi-directional treatment were also in line with the present literature [4, 5, 9].

PRGS is probably the best objective marker of local response and surrogate marker of overall survival available at the moment [10, 11]. The fact that at least four out of five patients where treatment response was monitored by PRGS had a score ≤2 at the third PIPAC procedure, also points towards a relevant patient selection. It is also interesting to note that the median survival was significantly higher in gastric cancer patients with PRGS response, defined according to a recent prospective study [10], compared to those without, and that we may expect similar results for other primary tumors (e.g. colon) when more long term follow up data become available in the database.

The inclusion of patients with PM from different primary tumors in prospective and randomized trials is important to elevate PIPAC directed therapy onto the next level [9]. In that aspect, it is somewhat surprising that only a quarter of the patients in this database had their PIPAC directed treatment as part of a prospective study.

As expected, disease progression was the most important reason (50 %) for stopping PIPAC treatment [12] but a more detailed evaluation of other reasons should be considered in future reports.

The lack of randomized trials makes PIPAC survival data from an international database especially interesting. Despite potential differences in patient selection and treatment among the reporting centers, the combination of large real-world data show encouraging median overall survival rates from first PIPAC treatment (Table 7). So far the present data represent one of the largest compilations of survival data from specific cancer populations treated by PIPAC and systemic chemotherapy [5].

The ISSPP PIPAC database has several limitations. Most important, more than 90 % of the procedures were reported by three centers, only, and the database mainly reflects the combined results from three major European PIPAC centers or group of institutions. Thus, the results may not be representative for centers having initiated their PIPAC program recently. The number of missing or incomplete date are substantial for some of the variables (e.g. date of death), and the relatively short observation time for patients alive is also important in relation to the presented survival data.

On the other hand, large real-world data are still important while awaiting randomized trials [3, 9], and the results from the database provides additional evidence supporting the present indications and details of PIPAC directed therapy. In addition, some of the limitations like incomplete data, understanding and reporting variables, reminders to users, as well as easy linking to the database and the recruitment of more centers worldwide are all potentially solvable problems that are prioritized by the ISSPP Registry group.

While recognizing these limitations it is also important to note that all international databases start with relative few centers, limited data and incomplete follow up. We believe that reporting even selected data may stimulate additional centers worldwide to actively participate in the ISSPP PIPAC database. In that aspect it is good news that several new PIPAC centers have started entering data in 2023 after deadline for this report, and they will be part of the next annual report, which is expected to include a substantial larger number of patients and procedures as well as a longer follow up. Provided that the ISSPP database is accepted and used by the PIPAC community it may provide the foundation for future research including IDEAL stage 4 reports [9].

### How to join the ISSPP PIPAC database

Access to the database requires an active ISSPP membership and registration with the database. More information and registration can be found on the ISSPP website (http://isspp.org/professionals/pipac-database/).

## Conclusions

The ISSPP PIPAC database provides substantial real-world data supporting the use of PIPAC directed therapy in patients with PM from different primary tumors. PIPAC is a safe procedure, and PIPAC directed therapy – mostly in combination with systemic chemotherapy, can induce objective local tumor response that may lead to prolonged survival in selected cancer patients. Contributions to the ISSPP PIPAC database so far are mainly from Europe, and the ISSPP Registry Group hopes that additional centers worldwide will join and include their patients in the database.
